# Spatio-temporal velocity variations observed during the pre-eruptive episode of La Palma 2021 eruption inferred from ambient noise interferometry

**DOI:** 10.1038/s41598-023-39237-9

**Published:** 2023-07-25

**Authors:** Iván Cabrera-Pérez, Luca D’Auria, Jean Soubestre, Monika Przeor, José Barrancos, Rubén García-Hernández, Jesús M. Ibáñez, Ivan Koulakov, David Martínez van Dorth, Víctor Ortega, Germán D. Padilla, Takeshi Sagiya, Nemesio Pérez

**Affiliations:** 1 Instituto Volcanológico de Canarias (INVOLCAN), Granadilla de Abona, 38600 Tenerife, Canary Islands Spain; 2 Instituto Tecnológico y de Energías Renovables (ITER), Granadilla de Abona, 38600 Tenerife, Canary Islands Spain; 3 Univ. Grenoble Alpes, Univ. Savoie Mont Blanc, CNRS, IRD, Univ. Gustave Eiffel, ISTerre, 38000 Grenoble, France; 4 Icelandic Meteorological Office, Reykjavík, Iceland; 5 Trofimuk Institute of Petroleum Geology and Geophysics SB RAS, Prospekt Koptyuga, 3, 630090 Novosibirsk, Russia; 6 Novosibirsk State University, Pirogova 2, 630090 Novosibirsk, Russia; 7 Institute of the Earth’s Crust SB RAS, Lermontova 128, Irkutsk, Russia; 8 Department of Theoretical Physics and Cosmos, Science Faculty, University of Granada, Avd. Fuenteneueva s/n, 18071 Granada, Spain; 9 Andalusian Institute of Geophysiscs, University of Granada, Campus de Cartuja, C/Profesor Clavera 12, 18071 Granada, Spain; 10 Disaster Mitigation Research Center, Nagoya University, Nagoya, Japan

**Keywords:** Geophysics, Seismology, Volcanology

## Abstract

On Sept. 19th, 2021, a volcanic eruption began on the island of La Palma (Canary Islands, Spain). The pre-eruptive episode was characterized by seismicity and ground deformation that started only 9.5 days before the eruption. In this study, we applied seismic interferometry to the data recorded by six broadband seismic stations, allowing us to estimate velocity variations during the weeks preceding the eruption. About 9.5 days before the eruption, we observed a reduction in the seismic velocities is registered next to the eruptive centers that opened later. Furthermore, this zone overlaps with the epicenters of a cluster of volcano-tectonic earthquakes located at shallow depth (< 4 km) and detached from the main cluster of deeper seismicity. We interpret the decrease in seismic velocities and the occurrence of such a shallow earthquake cluster as the effect of hydrothermal fluid released by the ascending magma batch and reaching the surface faster than the magma itself.

## Introduction

La Palma is one of the youngest islands among the volcanic archipelago of Canary Islands (Spain). On Sept. 19th, 2021, a volcanic eruption began on the island, which had a significant social and scientific impact. This eruption also had a catastrophic economic impact generating significant economic losses. The eruptive dynamics were mainly characterized by effusive phases interspersed with more explosive activity, during which eruptive columns dispersed ashes up to tens of kilometers away from the volcano.

The precursory phase of this eruption was characterized by intense volcano-tectonic seismicity, with magnitudes exceeding 4 M_L_ and hypocenters located at a depth of less than 10 km, together with ground deformation up to 16 cm on the vertical component of GPS stations. This phase lasted about a week and caught by surprise the scientific community for its short duration. However, given the large amount of scientific instrumentation (seismometers, GPS, etc.) operated by the Instituto Volcanológico de Canarias (INVOLCAN) and other scientific institutions, the entire pre-eruptive episode was accurately monitored and the civil protection authorities were notified in near real-time about the development of the volcanic unrest.

This work aims to detect seismic velocity variations during the pre-eruptive phase through seismic ambient noise interferometry and to compare these changes with the local seismicity detected before the eruption (D’Auria et al.^1^) and ground deformation. Seismic interferometry has been applied satisfactorily in different fields such as groundwater level^2,3^, fault zones^4^, the lunar environment^5^, geothermal exploration^6^, landslides monitoring^7^ and volcano monitoring. The first application of ambient noise interferometry to a volcano was realized by Sens‐Schönfelder and Wegler^5^, who observed velocity variations in Merapi volcano produced by changes in hydrological conditions. After this study, several investigations highlighted the effectiveness of the ambient noise interferometry method to monitor volcanoes^8–14^. The velocity variations observed before the eruptions generally consist of a reduction in the seismic velocity caused by the effect of the dilatation or compression of a part of the edifice resulting from the dynamics of the magma chamber^8,9^, pressurization of a magma pocket^10,14^, intrusion of magma^11,15^, topographic changes produced by a caldera collapse^12^ or to the effect of hydrothermal fluids^13,16^.

## Geological settings and the recent eruption

**Figure 1:**
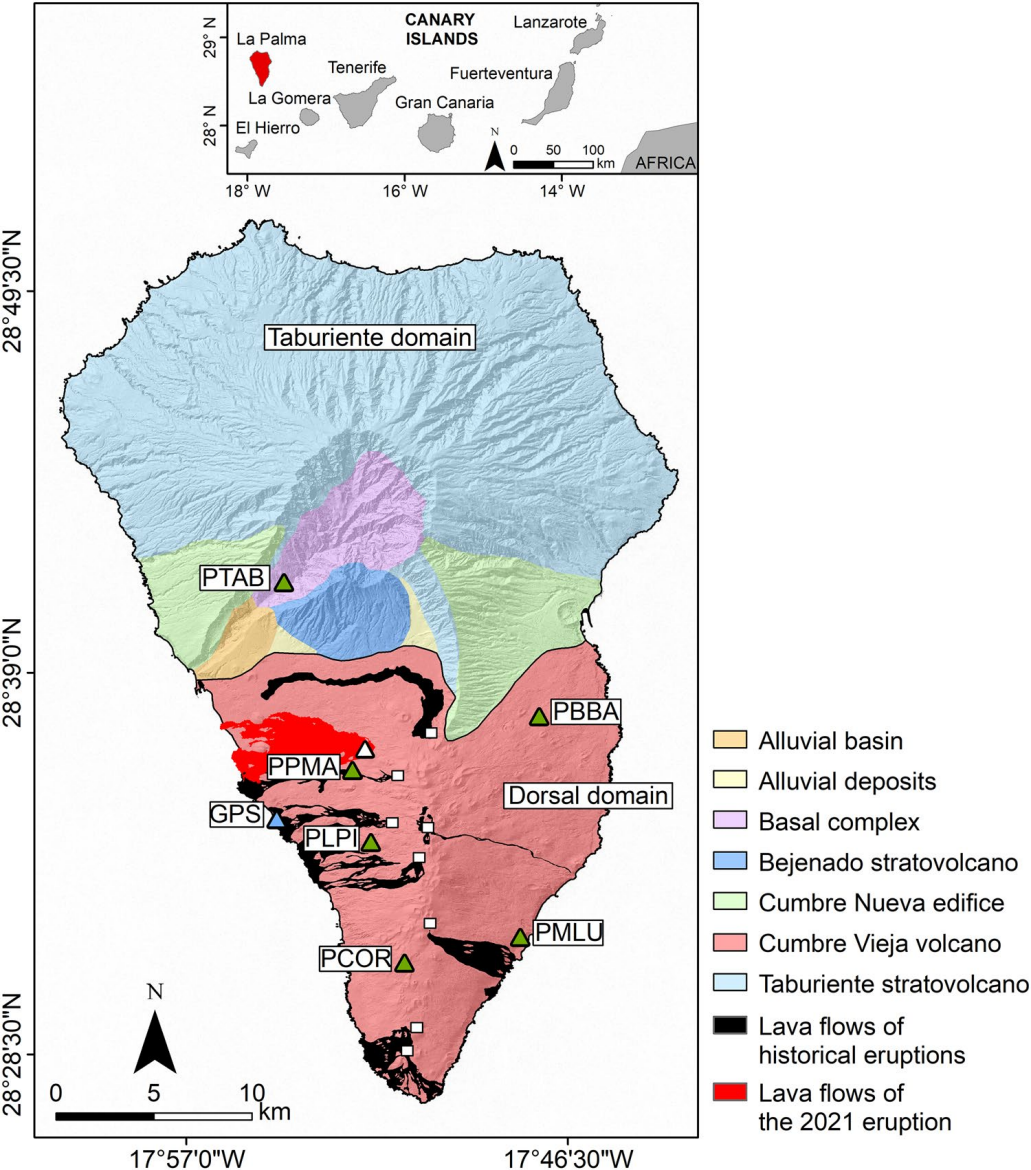
Geological map of La Palma island (modified from Padrón et al*.*^43^). The white triangle represents the location of the 2021 eruptive vent and white squares represent the location of historical eruptive vents. The blue and green triangles represent the location of the GPS ARID and seismic stations, respectively. The digital elevation model and historical lava flows were downloaded from the public graphic repository of GrafCan (http://www.grafcan.es). The 2021 lava flow was downloaded from the European agency Copernicus Emergency Management Service (https://emergency.copernicus.eu/mapping/list-of-components/EMSR546). The software used to generate this figure was QGIS 3.22 (https://www.qgis.org).

La Palma is located in the extreme NW of the Canary Islands. It is the third smallest island of the archipelago and one of the most active from a volcanological point of view, with eight historical eruptions in less than 600 years^17^. It is composed of two main geological domains: the Taburiente Domain and the Dorsal Domain (Fig. 1).

The Taburiente Domain is the oldest domain. It is located in the northern part of the island and it is composed of the superposition of stratovolcanoes with a semicircular base and a large depression in the central part (Caldera de Taburiente) (Fig. 1). This domain consisted of a submarine phase (4 Ma), represented by the Bassal complex, and a subaerial phase, which originated the big insular edifices conformed by Taburiente (from 1.77 to 1.20 Ma) and Bejenado (from 0.56 to 0.49 Ma) stratovolcanoes^18^.

The Dorsal Domain is more recent and currently volcanically active. It is located in the southern part of the island, south of the Taburiente Domain. This volcanic ridge has a North–South orientation and an elongated shape. It is divided into two sectors: in the northern sector is the Cumbre Nueva with an arched shape, while in the southern sector is the Cumbre Vieja with a North–South direction and an extension of 21.5 km. Its formation began 0.123 Ma ago and continues with a high volcanic activity until today^18^. This domain hosted seven historical eruptions, including the most recent 2021 eruption (Fig. 1).

The last 2021 eruption of La Palma was announced in 2017, by the first appearance of seismicity^19^. The background seismicity of the island was practically non-existent during the last decades, as reflected in the seismic catalogues of the Instituto Geográfico Nacional (IGN). Conversely, between 2017 and 2021, nine seismic swarms took place on the island, with approximately 700 earthquakes located beneath Cumbre Vieja sector at depths between 10 and 20 km. This seismicity was located under the Cumbre Vieja volcano. The 2021 pre-eruptive unrest started on Sept. 11st, only 9.5 days before the eruption (Fig. 3C). During this episode, seismicity quickly migrated from a depth of 10 km to the surface, following the ascending path of the magma^1^. On Sept. 15th, we observed earthquakes located at very shallow depth (< 4 km) and detached from the main seismicity cluster which was located at depths of 6–8 km (Fig. 3C). A very energetic co-eruptive volcanic tremor also began with the eruption onset on Sept. 19th.

## Methodology and data processing

The seismic data used in this work come from the Red Sísmica Canaria (C7) operated by INVOLCAN (Instituto Volcanológico de Canarias, 2016). We used recordings from six broadband seismic stations (Nanometrics © Trillium Compact 120 s and Güralp ©, 3ESPC Series) with a sampling rate of 100 Hz (Fig. 1). The time range used for the analysis covers the interval from Aug. 1st to Sept. 25th, 2021. We analysed the data using the MSNoise python package^19^ to estimate relative velocity variations. This software has been applied successfully in different studies of ambient noise interferometry^14,20–23^.

### Estimation of the relative velocity variations

The procedure to estimate relative velocity variations ($dv/v$) has been carried out using the following workflow. Recorded data were downsampled to 20 Hz, bandpass filtered in the 0.1–1.0 Hz frequency range, and pre-processed applying spectral whitening followed by one-bit temporal normalization^24^. Then, we computed the cross-correlation of ambient noise recordings among pairs of stations to obtain the empirical seismic Green’s Functions (GFs), using the vertical–vertical (ZZ) components. To estimate $dv/v$ it is necessary to compare the coda of the obtained GFs with a Reference Green’s Function (RGF), which has been computed stacking over the first twenty days of the data in our case (from Aug. 1st to 20th). Assuming a relative velocity variation $dv/v$ in a homogeneous space, one can prove that^25^:

1 $$\frac{dv}{v}=-\frac{d\tau }{\tau },$$

where $d\tau$ represents the measured time delay and $\tau$ the traveltime. Actually, there are two methods to extract $dv/v$ from the empirical GFs: the stretching technique (Sens-Schönfelder and Wegler^25^) and the moving window cross-spectral analysis (MWCSA)^26–28^. In both methods, the $dv/v$ is estimated using the GFs part corresponding to the scattered wavefield at different time lags. Duputel et al.^9^ showed that both methods provide similar results and therefore concluded that both approaches are equivalent. However, Clarke et al.^29^ demonstrated that the MWCSA method is more efficient to detect very small $dv/v$. For this reason, in this study, we use the MWCSA method. The error of this estimation can be determined using the squared misfit of the modeled slope of the linear regression of the time-delay ($d\tau$) measurements^29^.

For each day, we computed cross-correlations on two minute-long windows, which were subsequently stacked over the previous 5 days. The use of shorter stacking windows led to excessive uncertainty over the retrieved $dv/v$ values. Then, we compared GFs with the RGF using the aforementioned MWCSA method on five-second-long windows and a step of two-second-long over the whole 240 s (− 120 s ÷ 120 s) of the cross-correlation functions to estimate a value of $dv/v$**.** This window length was selected as being the best compromise between resolution and uncertainty. Figure S1 in the supplementary materials shows three examples of interferograms for station pairs PLPI-PPMA, PLPI-PCOR and PPMA-PCOR (Fig. 1) within the 0.1–1.0 Hz range. We can observe that after the start of the eruption, the GFs show an erratic shape, where the causal and acausal parts are not correctly defined. This is a consequence of the volcanic tremor, which started just at the beginning of the eruption. The tremor acts as a source of contamination due to a persistent coherent signal with a localized source in the 0.3–4.0 Hz frequency range, which encompasses the frequency range of our study (see Fig. S2 in the supplementary materials). For this reason, we decided to limit our interpretation of *dv/v* values until the start of the eruption. All the daily $dv/v$ for all the pairs of stations are shown in Fig. S3 in the supplementary materials.

### Spatial distribution of $dv/v$

In order to determine the spatial distributions of $dv/v$ we applied a linear inversion technique. We used the analytical approach of Del Pezzo and Ibáñez^30^ to calculate the sensitivity kernels for the propagation of scattered waves between each station pair:

2 $$\begin{aligned} {K}_{num}(x,y,{x}_{i},{y}_{i},{x}_{j},{y}_{j} ) & =\frac{1}{{6\pi (D\delta )}^{2}}exp\left(-\left(\frac{{\left(x-\frac{{x}_{i}+{x}_{j}}{2}\right)}^{2}}{{2\left(\delta D\right)}^{2}}+\frac{{\left(y-\frac{{y}_{i}+{y}_{j}}{2}\right)}^{2}}{{2\left(\delta D\right)}^{2}}\right)\right) \\ & \quad + \frac{1}{{2\pi (\delta D)}^{2}} exp\left(-\left(\frac{{\left(x-{x}_{i}\right)}^{2}}{{2\left(\delta D\right)}^{2}}+\frac{{\left(y-{y}_{i}\right)}^{2}}{{2\left(\delta D\right)}^{2}}\right)\right) \\ & \quad + \frac{1}{{2\pi (\delta D)}^{2}} exp\left(-\left(\frac{{\left(x-{x}_{j}\right)}^{2}}{{2\left(\delta D\right)}^{2}}+\frac{{\left(y-{y}_{j}\right)}^{2}}{{2\left(\delta D\right)}^{2}}\right)\right), \end{aligned}$$

where ($${x}_{i},{y}_{i} )$$ and ($${x}_{j},{y}_{j} )$$ represent the coordinates of the (virtual) sources and receivers, $$\delta$$ represents the spatial aperture of the weighting function and $$D$$ represents the source-receiver distance. Figure S4 in the supplementary materials shows an example of sensitivity kernel for the station pair PCOR-PLPI (Fig. 1). Del Pezzo and Ibáñez^30^ used this kind of kernel for imaging the spatial distribution of the intrinsic attenuation parameter Q. However, this formulation can be useful for imaging $$dv/v$$ as well, being both quantities related to the scattered wavefield. The kernel of Del Pezzo and Ibáñez^30^ assumes diffusion as a scattering regime. Since we computed the $$dv/v$$ overtime windows of 120 s, which is many times the ballistic travel-time for our network, we conclude that this assumption is correct in our case. The authors suggested using a value of 0.2 for the parameter $$\delta$$. Using this kernel we can express the observed $$dv/v$$ for a station pair (s_i_,s_j_) as:

3 $$\frac{dv}{v}\left({s}_{i},{s}_{j}\right)={n}^{-1}{\int }{\int }{K}_{num}(x,y,{x}_{i},{y}_{i},{x}_{j},{y}_{j} )\frac{dv}{ v}\left(x,y\right)dx dy$$

with n being a normalization factor:

4 $$n={\int }{\int }{K}_{num}(x,y,{x}_{i},{y}_{i},{x}_{j},{y}_{j} ) dx dy$$

We discretize this forward problem by representing the continuous function $dv/v$ as a mesh of 19 × 27 km regular tiles having a size of 1.4 × 1.8 km^31^. The supplementary materials show the ray path and the 2D kernel density map in Figs. S5 and S6, respectively. The resulting discrete inverse linear problem was solved using the Truncated Singular Value Decomposition, selecting the appropriate number of eigenvalues with the L-curve approach^32^.

## Results

**Figure 2:**
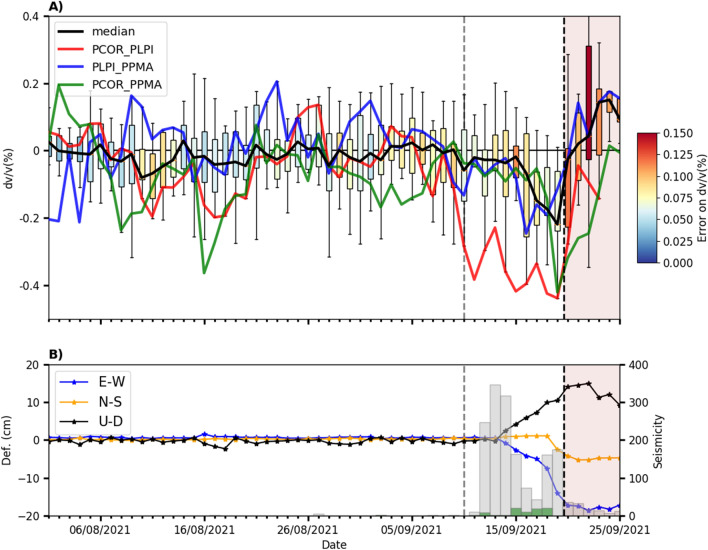
Comparison of daily $dv/v$ with the seismicity and deformation produced during the pre-eruptive and eruptive periods (vertical black dashed line showing the eruption onset). The vertical gray dashed line represents Sept. 10th. (**A**) Statistical analysis of daily $dv/v$ for all the station pairs (median, black line), together with some $dv/v$ for specific station pairs (PCOR_PLPI: red line, PLPI_PPMA: blue line, PCOR_PPMA: green line). Each boxplot represents the minimum and maximum values of $dv/v$ (lower and upper horizontal lines), its lower and upper quartiles (lower and upper box limits), and its median. The color of the boxplots represents the estimated error on $dv/v$. (**B**) Time series of GPS ARID deformation appear as blue, orange and black lines for the E-W, N-S and U-D components, respectively. The histogram bars indicate the seismicity possibly related to the fluid injection (green dots) and magmatic intrusion (black dots). The relative velocity variation curves were obtained using MSNoise software^19^ (http://www.msnoise.org).

**Figure 3:**
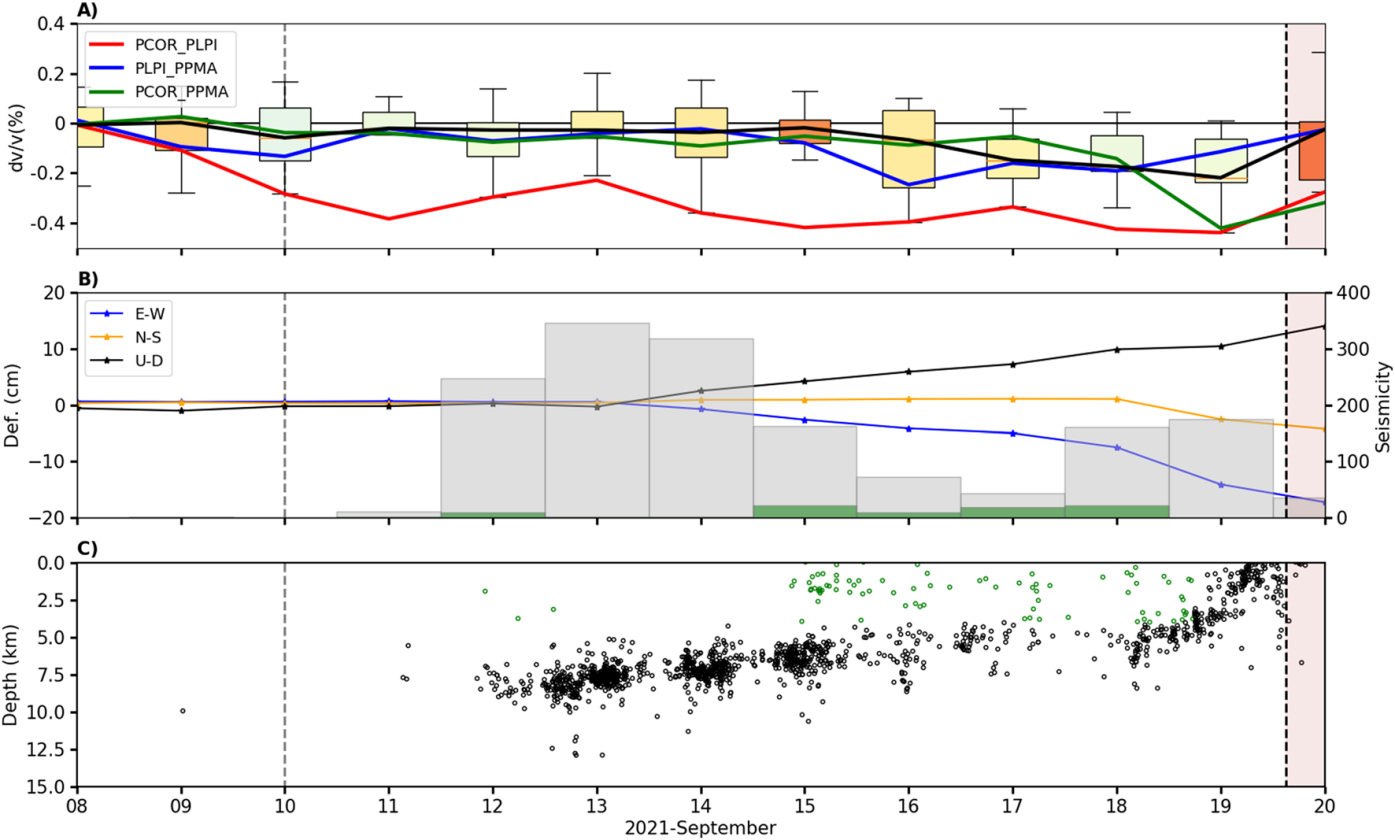
The temporal window of Fig. 2 has been zoomed in to highlight the comparison of $dv/v$ with seismicity and deformation between Sept. 8th and 19th. This time window encompasses the pre-eruptive period. (**A**) Daily $dv/v$ for all station pairs (median, black line) and specific station pairs (red, blue and green lines). (**B**) Deformation times series (GPS ARID) and seismicity histograms. (**C**) Depth distribution of the seismicity related to the fluid injection (green dots) and magmatic intrusion (black dots).

Figure 2A shows the daily seismic velocity variations corresponding to the median of all the station pairs (Fig. 2A, black line) and specific station pairs (Fig. 2A, coloured lines) from Aug. 1st to Sept. 25th. The median time series does not show significant velocity variations at the beginning, with mean values generally remaining within ± 0.01% until Sept. 10th (Fig. 2A, black line). Since Sept. 10th, $dv/v$ started decreasing evidently in the PCOR_PLPI pair, reaching a minimum of − 0.4% on Sept. 18th (Figs. 2A and 3A). Between Sept. 18th and 19th, the average $dv/v$ attains a minimum with an average value of − 0.21% (Figs. 2A and 3A). We note that the days in which significant variations on the average $dv/v$ are observed the error values are generally lower than 0.075% (color-coded in Figs. 2A and 3A).

**Figure 4:**
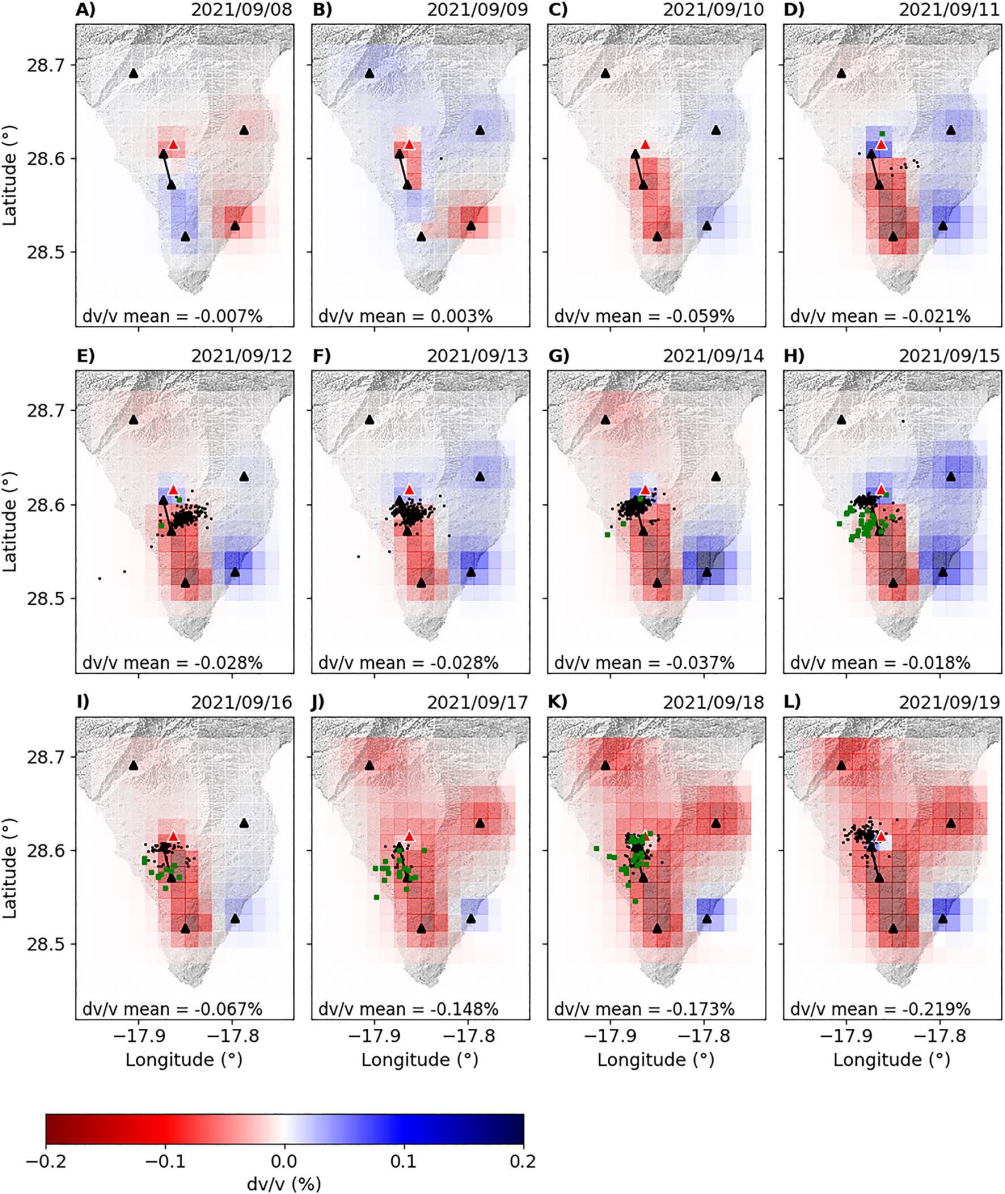
Spatial distribution of $dv/v$ for different dates in September 2021. The green and black dots represent the seismicity related to the fluid injection and magmatic intrusion, respectively. Seismic stations appear like black triangles, and a red triangle shows the 2021 eruptive vent. The black line represents the approximate raypath of the station pair PLPI-PPMA, which is the closest to the eruption site. The digital elevation model was downloaded from the public graphic repository of GrafCan (http://www.grafcan.es).

Figure 4 shows the results of the spatial mapping of daily $dv/v$ from Sept. 8th to 19th. The spatial distribution of $dv/v$ between Sept. 8th and 9th shows low $dv/v$ values in the eruption zone and in the eastern part of Cumbre Vieja (Fig. 4A and B). We consider these low $dv/v$ values as artifacts produced during the inversion process because we don’t have enough resolution to observe such minor anomalies. Similar anomalies considered as artifacts are observed in the month prior to the eruption (see Fig. S8 of supplementary materials). On Sept. 10th, we observe low $dv/v$ values located in the southern part of the eruption site, with an average value of − 0.059% (Fig. 4C). During this day, no seismicity was recorded and no deformation was observed (Figs. 2B and 3B). Between Sept. 11th and 14th, the $dv/v$ values observed in this zone with the station pair PLPI-PCOR (Fig. 1) decreased, reaching − 0.38% in Sept. 14th (Fig. 3A). During this period, a deep seismic swarm (> 4 km) was recorded and deformation began to occur (Figs. 2B and 3B). Between Sept. 15th and 16th, the average $dv/v$ started to decrease, reaching − 0.41% in some station pairs (Fig. 3A). We started recording shallow earthquakes (< 4 km) during these days, and the deformation continued increasing. On Sept. 17th, there was a generalized decrease of the $dv/v$ values in most of the station pairs (Fig. 4J), with an average $dv/v$ value of − 0.148%. This generated a much larger anomaly distribution, encompassing most of the Cumbre Vieja volcanic complex. Between Sept. 18th and 19th, the $dv/v$ values continued decreasing, reaching − 0.43% in some station pairs (Fig. 3B).

## Discussion and conclusion

The most important result of this work is the relevant decrease of $dv/v$ observed 9.5 days before the eruption onset. This decrease started on Sept. 10th, the day before the onset of the seismicity. Between Sept. 10th and 14th, the $dv/v$ continued decreasing. During this period, the deep seismicity (> 4 km) continued increasing and ground deformation started to be recorded on Sept. 14th. Then, on the 15th of September, the values of *dv/v* were still decreasing and a shallow seismicity (< 4 km) was observed (Fig. 3C). This seismicity was distributed between 1 and 5 km south of the eruptive center (Fig. 4). Between the 12th and 15th of September, we observed a velocity increase in the southeastern part of the island. The most likely explanation is that this increase in velocity is related to negative volumetric strain (compression) due to the ground deformation.

We exclude the stress/strain field variation in the volcanic edifice as a dominant mechanism to explain the observed decrease in $dv/v$, due to the lack of significant ground deformation between Sept. 10th and 14th. Actually, we observe that the station pair showing the most evident decrease (PCOR_PLPI) is located to the south of the area of the eruptive vent, where the highest ground deformation was observed^33^ (Fig. 4). Furthermore, we discard the effect of ground shaking produced by earthquakes as a causative mechanism for the velocity drop, as the earthquakes that occurred during this period had a magnitude generally lower than 2.5 M_L_ (see Fig. S7 of supplementary materials), their hypocenters were deeper than 5 km (Fig. 3C) and their frequency content was above the higher limit of 1 Hz considered for $dv/v$ estimations (Fig. S2). Moreover, the most important velocity drop occurs a few kilometers to the south of the area of most intense seismicity (Fig. 4C–I). Another possible mechanism which can be invoked to justify the velocity drop is the magmatic intrusion itself, with the associated fracturing process. Again, we consider this mechanism unlikely before Sept. 19th since the hypocenter depths (Fig. 3C) clearly show that the magma reached shallow depths (< 4 km) only the day before the eruption. Considering the velocity model of D’Auria et al.^1^ for the given range periods used in the analyses (1.0–10.0 s), the penetration depth of the Rayleigh waves is just a few kilometers (see Fig. S9 of supplementary materials). Therefore, we can exclude the direct involvement of magma in the process since, as also testified by the hypocenter depths (Fig. 3C), the magma reached the surface only on the day of the eruption (19th of Sept.). Moreover, this explanation is also not compatible with the fact that the most relevant velocity variations are located a few kilometers to the south of the eruptive vents (Fig. 4). However, the marked drop of dv/v observed on the day before the eruption could be related to the magmatic intrusion reaching the surface.

**Figure 5:**
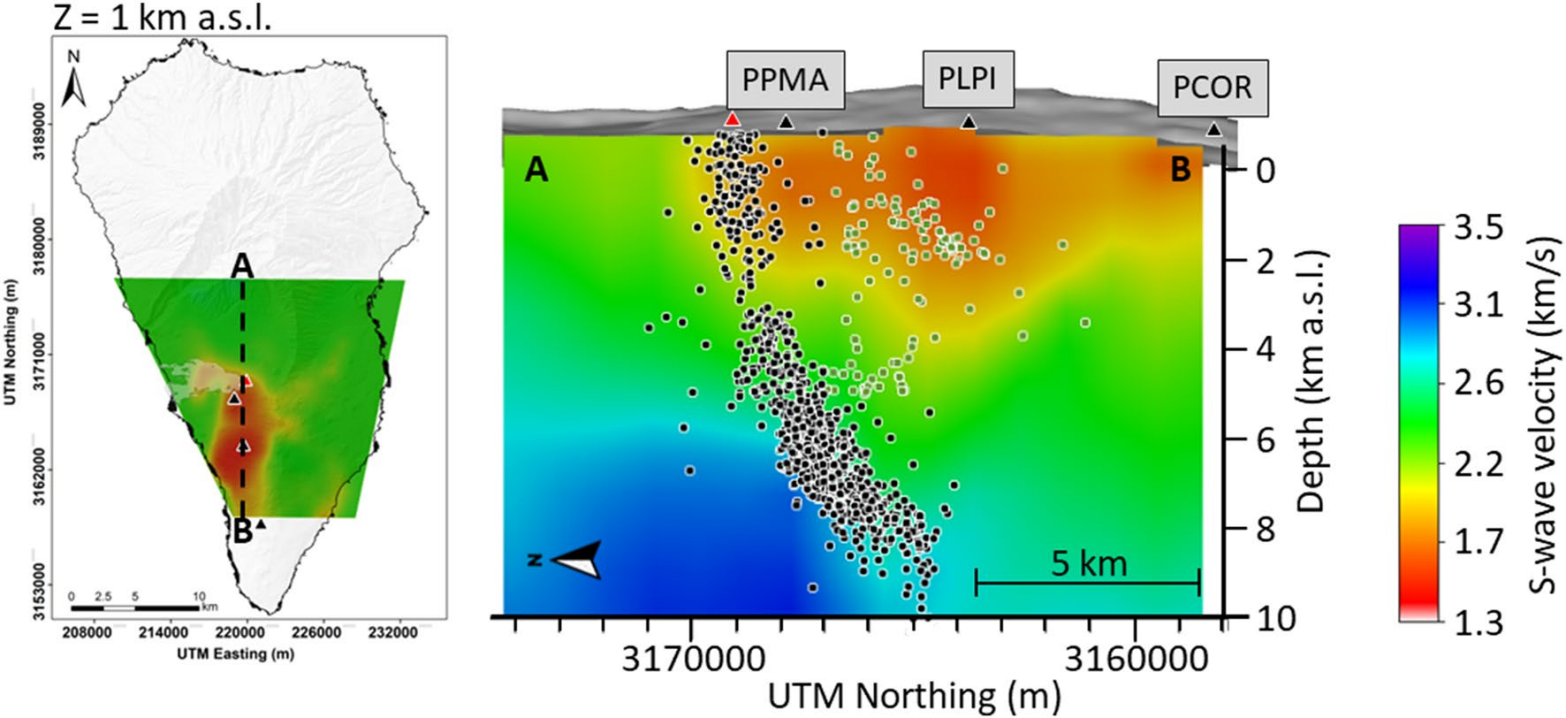
Horizontal (left) and Vertical N-S cross-sections (cf. A-B in left figure) of the 3D S-wave velocity model obtained by D’Auria et al.^1^. The green and black dots represent the seismicity related to the fluid injection and magmatic intrusion, respectively.

Thus, we consider that the observed velocity drop can be explained by the ascent of hydrothermal fluids towards the surface through areas of weakness, such as those imaged in the Vs model obtained by D'Auria et al.^1^ (Fig. 5) and the resistivity model of Di Paolo et al.^34^ (see Fig. 2C of Di Paolo et al.^34^). Both models show that this area could have hosted a hydrothermal reservoir prior to the eruption, at a depth of about 2 km b.s.l. The area affected by the decrease in dv/v is mostly located between the station PLPI and PCOR, and extends approximately between 2 and 12 km south of the eruptive center (Fig. 4), coinciding with the previously identified hydrothermal reservoir.

The source of these hydrothermal fluids can be ascribed to the ascending magma batch itself. The upward migration of hypocenters and the increase in the ground deformation clearly suggest that the magma was rising at least since Sept. 13th. The consequent depressurization of the magma must have produced the exsolution and the release of the dissolved gases, which migrated upward through fracture systems faster than the magma itself. Recent petrological observations realized by Pankhurst et al.^35^ determined that the magmas emitted during the initial phase of the eruption were more hydrated, as evidenced by the presence of amphibole^36^. This mineral disappeared from the emitted products during the later phases of the eruption, testifying a lower water content. This supports our hypothesis about the pressurization of a shallow hydrothermal system by the injection of gases released by the ascending magma at depth. Note that a decrease in the average seismic velocities due to the input of hydrothermal fluids is already documented in the scientific literature^13,16,37^. The same holds for the triggering of earthquakes caused by the injection of hydrothermal fluids^38^. In Fig. 5 we represent a north–south cross-section of the S-wave tomographic velocity model from D’Auria et al.^1^. It can be observed that the horizontal extent of a low-velocity anomaly (map on the left side of Fig. 5), which has been interpreted as a hydrothermal reservoir, coincides with the area of greater velocity decrease before the eruption (Fig. 4). From this figure, it is also clear that the hypocenters, which we attribute to the injection of hydrothermal fluids, are located on the northernmost side of this reservoir. Therefore we conclude that fluid-induced earthquakes are located only within the zone where hydrothermal fluids, exolving from the magma, are injected into the reservoir. This possibly occurs because of the stronger fluid pressure gradients associated with this area.

From Fig. 3C, we observe that the earthquakes (an therefore the magma) approach quickly the surface between 18 and 19th of Sept. Therefore, as we mentioned before, the mechanism that caused the decrease of $dv/v$ the day before the eruption can be strongly affected by the magmatic intrusion. The intrusion of magma at shallow depth generates structural damage and elastic strain changes in the crust which could explain the rapid drop of $dv/v$. The decrease in the average seismic velocities due to magmatic intrusion is already documented in the scientific literature^15,39^.

The results of our analysis demonstrate once again the usefulness of ambient noise interferometry as a volcano monitoring tool. The sensitivity of this method in detecting velocity variations related to volcanic processes and, in particular, to magmatic or hydrothermal fluid injections, makes it a valuable tool for better understanding the volcano dynamics. In our case, it was fundamental to correctly interpret the swallow seismicity observed, 9.5 days before the eruption onset. A major drawback of this technique is that it is negatively affected by coherent sources like a volcanic tremor. For this reason, this method was not applied for the syn-eruptive monitoring of La Palma 2021 eruption.

## Supplementary Information

Supplementary Figures.

## Data Availability

All data generated or analysed during this study are included in this published article are in the Zenodo repository, 10.5281/zenodo.6678861.
