# Supplementary material for: Spatio-temporal velocity variations observed during the pre-eruptive episode of La Palma 2021 eruption inferred from ambient noise interferometry
Source: Sci Rep. 2023 Jul 25;13:12039. doi: 10.1038/s41598-023-39237-9 (PMC10368664; doi:10.1038/s41598-023-39237-9)
Supplement: Supplementary file 1 — Supplementary Figures. [file 41598_2023_39237_MOESM1_ESM.pdf]

Scientific Reports

Supporting Information for

[Spatio-temporal velocity variations observed during the pre-eruptive episode of La Palma 2021 eruption inferred from ambient noise interferometry.]

Iván Cabrera-Pérez[1] , Luca D'Auria[1,2] , Jean Soubestre[3,4], Monika Przeor[1] , José Barrancos[2] , Rubén García-Hernández[1], Jesús M. Ibáñez[8,9], Ivan Koulakov[5,6,7], David Martínez van Dorth[1] , Víctor Ortega[1], Germán D. Padilla[2] , Takeshi Sagiya[10] and Nemesio Pérez[1,2]

[1] Instituto Volcanológico de Canarias (INVOLCAN), 38600 Granadilla de Abona, Tenerife, Canary Islands, Spain. E-mail: ivan.cabrera.perez1@gmail.com

[2] Instituto Tecnológico y de Energías Renovables (ITER), 38600 Granadilla de Abona, Tenerife, Canary Islands, Spain
[3] Univ. Grenoble Alpes, Univ. Savoie Mont Blanc, CNRS, IRD, Univ. Gustave Eiffel, ISTerre, 38000 Grenoble, France
[4] Icelandic Meteorological Office, Reykjavik, Iceland
[5] Trofimuk Institute of Petroleum Geology and Geophysics SB RAS, Prospekt Koptyuga, 3, 630090 Novosibirsk, Russia
[6] Novosibirsk State University, Novosibirsk, Russia, Pirogova 2, 630090 Novosibirsk, Russia
[7] Institute of the Earth's Crust SB RAS, Lermontova 128, Irkutsk, Russia
[8] Department of Theoretical Physics and Cosmos. Science Faculty. Avd. Fuenteneueva s/n. University of Granada. 18071. Granada. Spain.
[9] Andalusian Institute of Geophysiscs. Campus de Cartuja. University of Granada. C/Profesor Clavera 12. 18071. Granada. Spain.

[10]Disaster Mitigation Research Center - Nagoya University, Nagoya, Japan

Contents of this file

Figure S1 shows interferograms for three pairs of stations
Figure S2 shows the spectral width of the network covariance matrix
Figure S3 shows the daily dv/v for all the pairs of stations
Figure S4 shows an example of the 2D Kernel for a specific receiver source
Figure S5 shows the raypath for all the pairs of stations
Figure S6 shows the 2D kernel density map for all the pairs of stations
Figure S7 shows the temporal distribution of magnitudes during the pre-eruptive phase
Figure S8 shows the spatial distribution of dv/v for different dates in August 2021
Figure S9 shows the sensitivity kernels for different periods

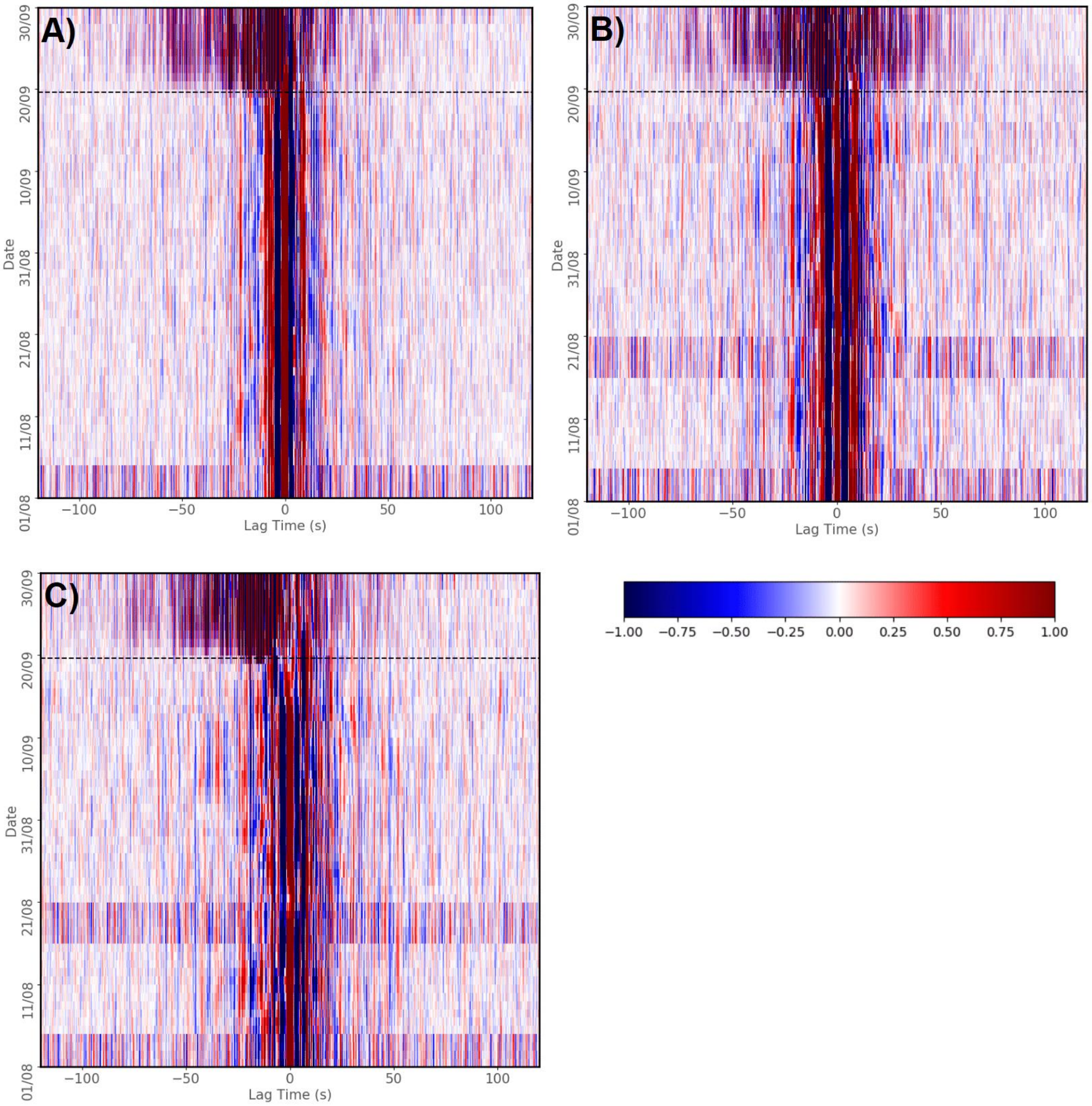

Figure S1. Interferograms of station pairs A) PLPI-PPMA, B) PLPI-PCOR and C) PPMA-PCOR in the 0.1 - 1.0 Hz frequency range. The black dashed line represents the eruption onset.

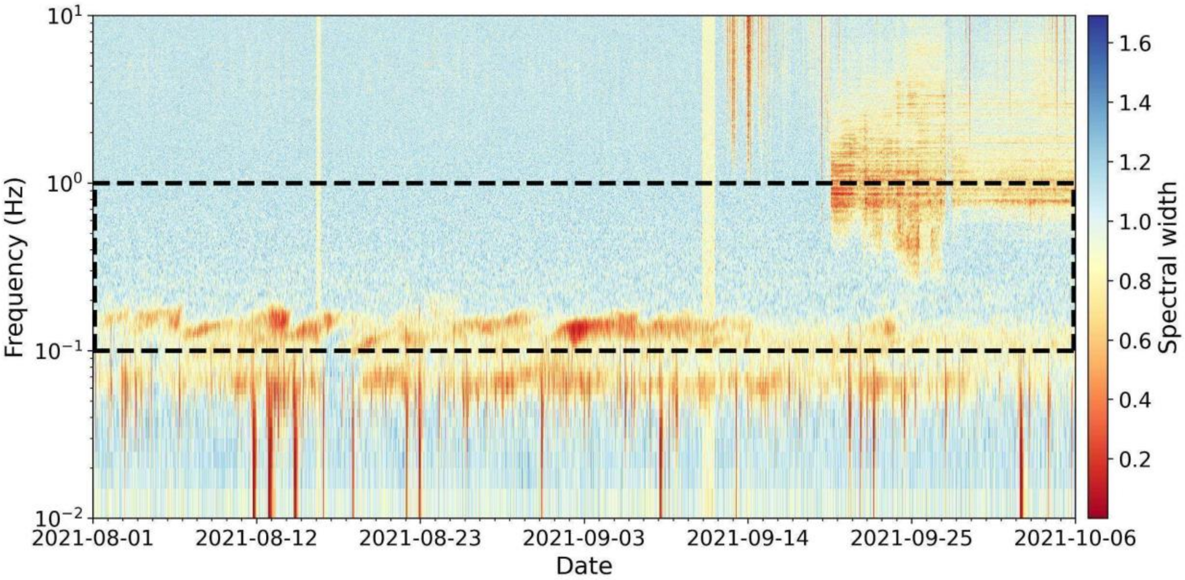

Figure S2. The spectral width represented in this time-frequency plot is a proxy of the number of sources composing the recorded wavefield, with low spectral width value corresponding to a dominant source and high value corresponding to seismic noise. It is estimated from a network covariance matrix analysis (Seydoux et al., 2016). The black dashed rectangle represents the frequency range in which we computed the cross-correlation of the ambient noise.

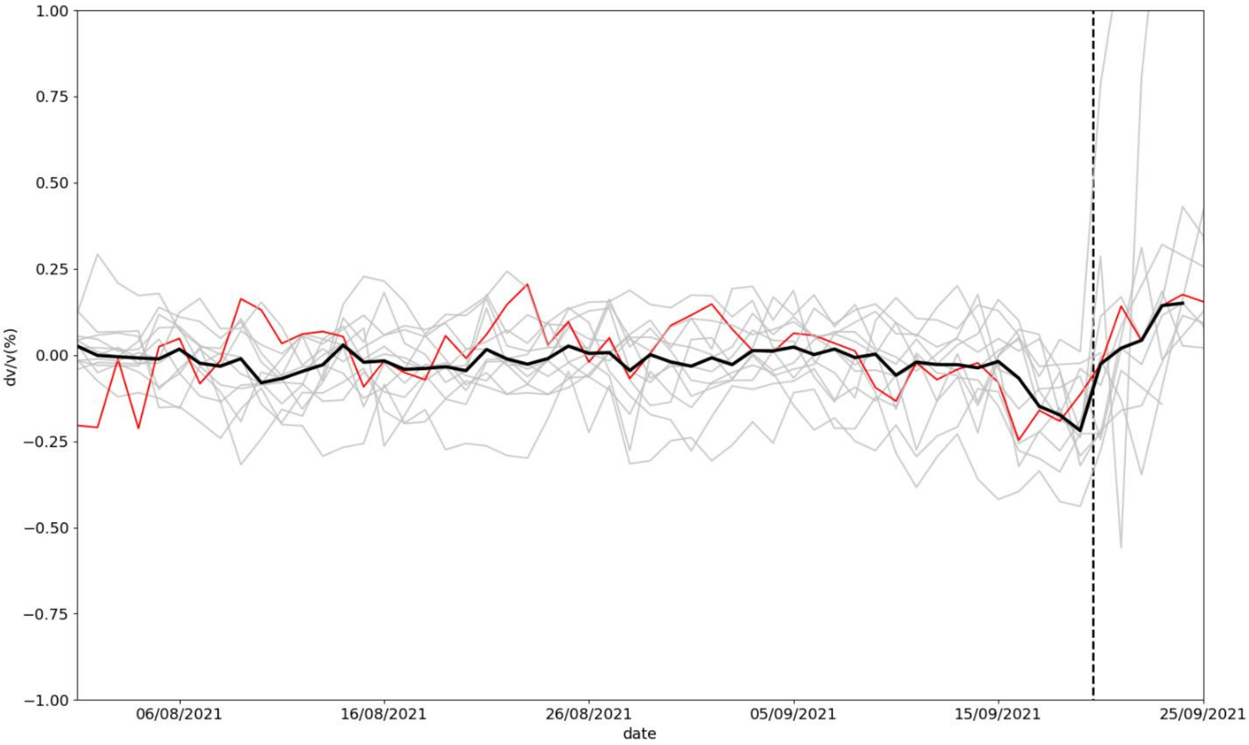

Figure S3. Daily dv/v for all the pairs of stations (gray lines). The red and black lines represent the closest station pair to the eruption site (PLPI-PPMA) and the mean, respectively.

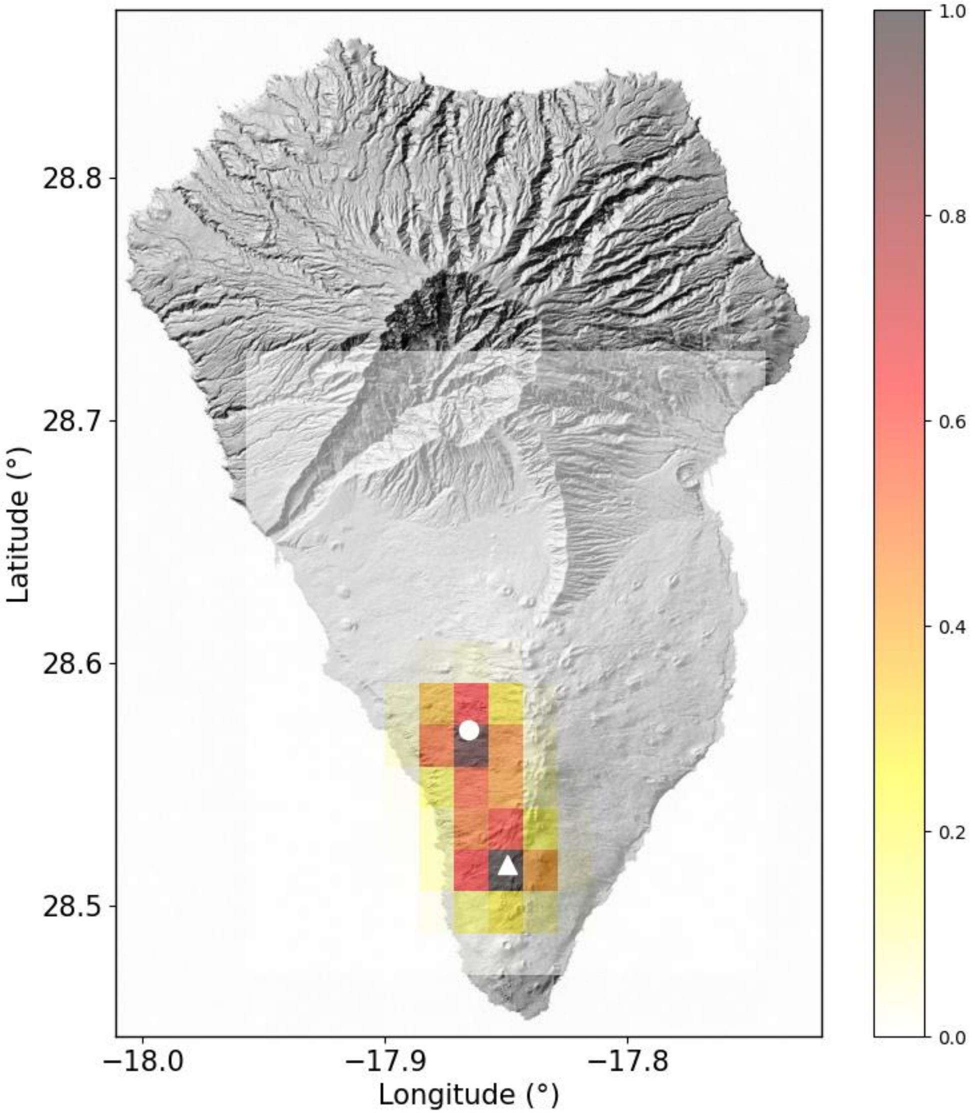

Figure S4. Example of 2D Kernel (equation (2) in the main text) for the station pair PLPI-PCOR (white circle and triangle, respectively, cf. Fig. 1). The digital elevation model was downloaded from the public graphic repository of GrafCan (www.grafcan.es).

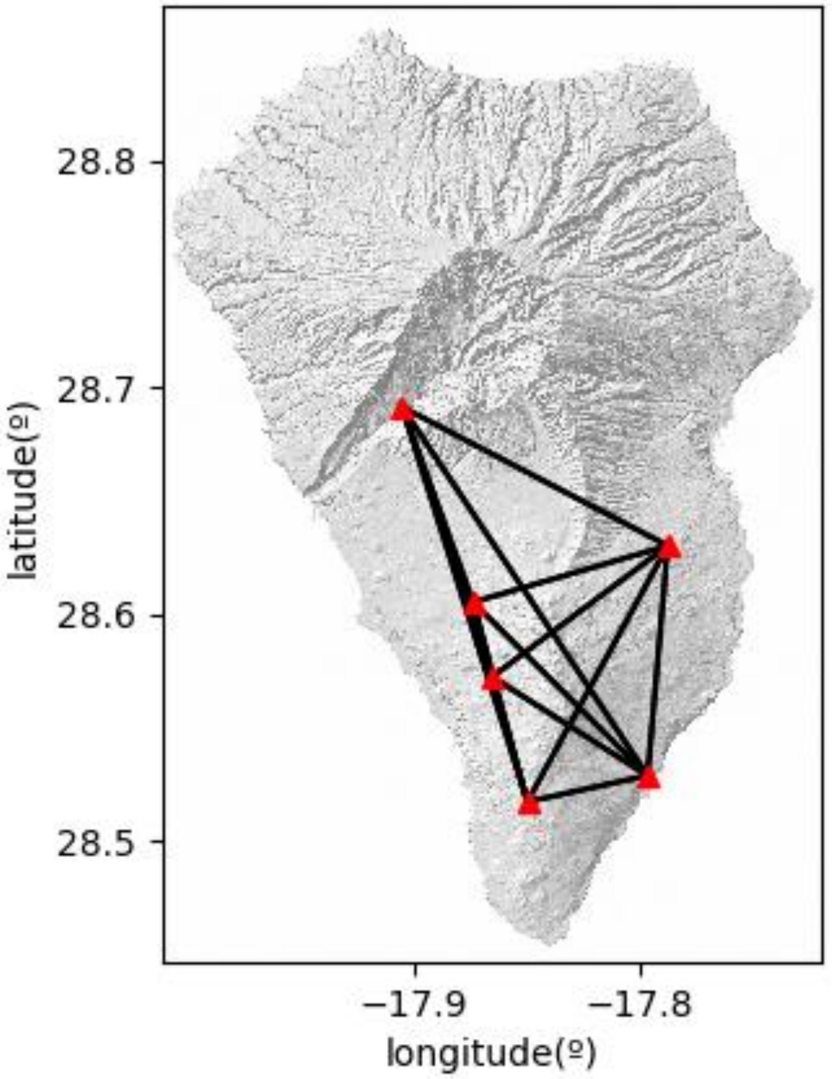

Figure S5. Raypath map of the seismic network. The red triangles represent the seismic stations and the black lines are the raypath for all the pairs of stations. The digital elevation model was downloaded from the public graphic repository of GrafCan (www.grafcan.es).

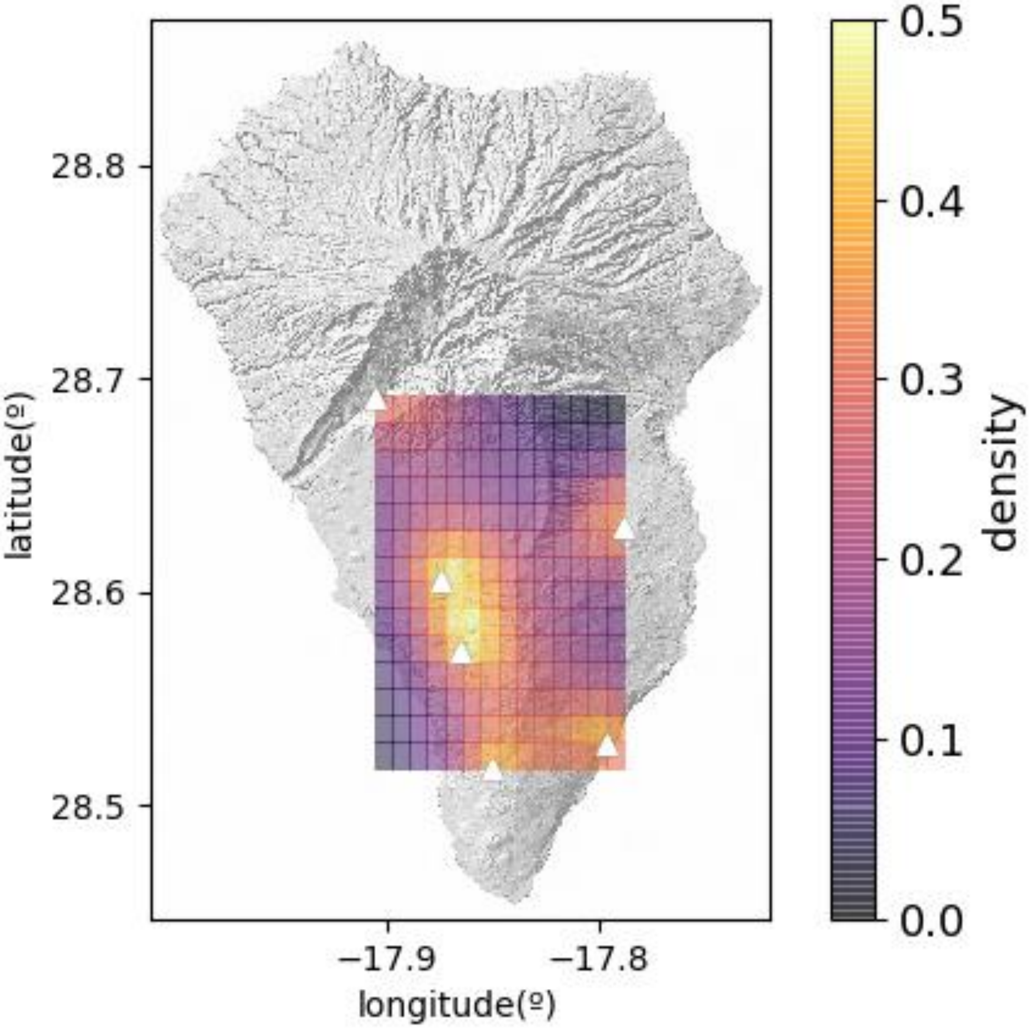

Figure S6. Density map obtained using the 2D sensitivity kernels for all the pairs of stations. The digital elevation model was downloaded from the public graphic repository of GrafCan (www.grafcan.es).

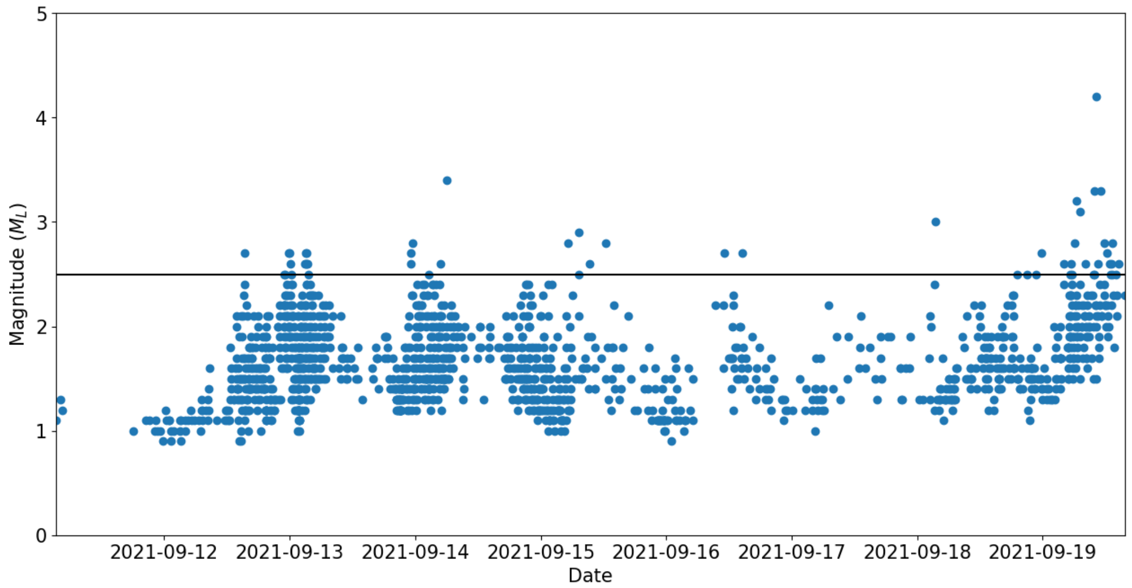

Figure S7. Temporal distribution of magnitudes during the pre-eruptive phase.

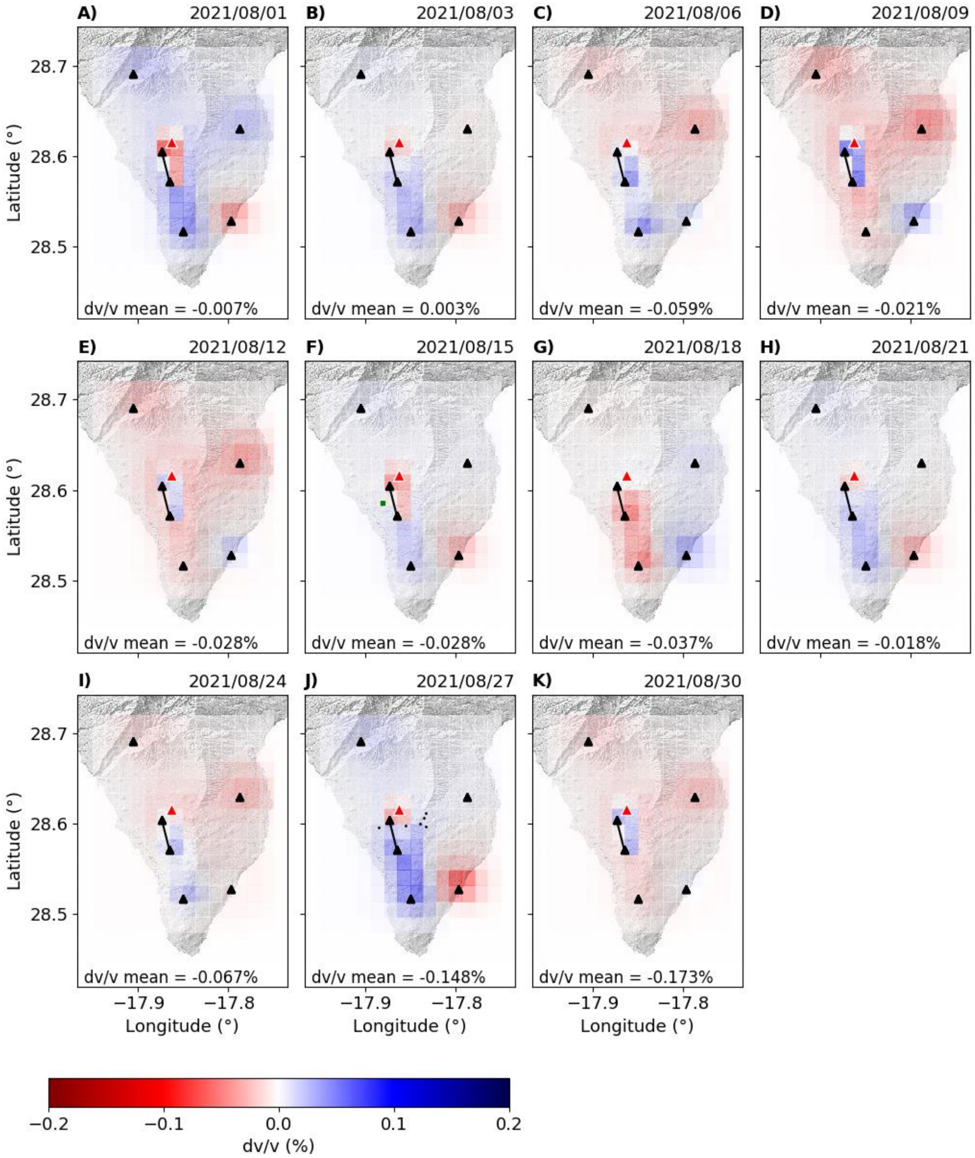

Figure S8. Spatial distribution of dv/v for different dates in August 2021. The green and black circles represent the location of earthquakes shallower and deeper than 4 km, respectively. Seismic stations appear like black triangles, and a red triangle shows the 2021 eruptive vent. The black line represents the approximate raypath of the station pair PLPI-PPMA, which is the closest to the eruption site. The digital elevation model was downloaded from the public graphic repository of GrafCan (www.grafcan.es).

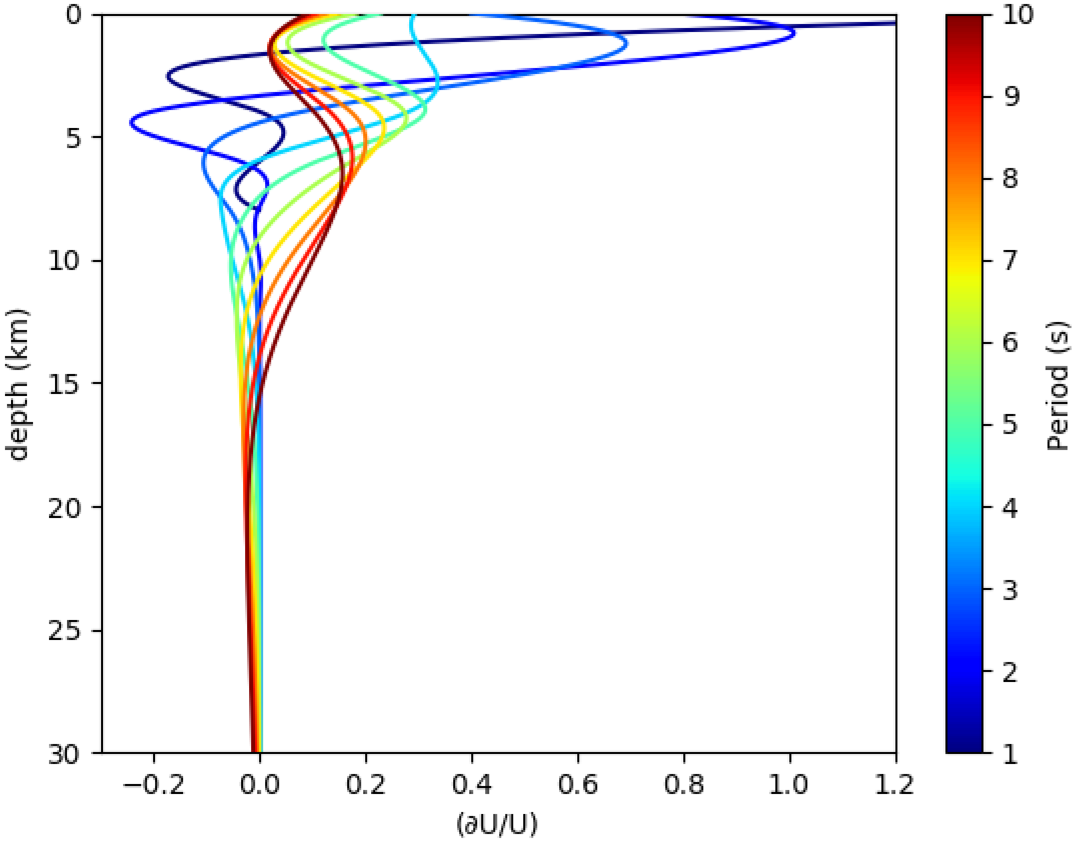

Figure S9. Fundamental mode of the Rayleigh wave group velocity sensitivity kernel for periods between T = 1 s and T = 10 s. The kernel is based on the 1-D mean S-wave velocity model obtained by D'Auria et al. [1].
